# Modification of Multiwalled Carbon Nanotubes and Their Mechanism of Demanganization

**DOI:** 10.3390/molecules28041870

**Published:** 2023-02-16

**Authors:** Yuan Zhou, Yingying He, Ruixue Wang, Yongwei Mao, Jun Bai, Yan Dou

**Affiliations:** 1 School of Water and Environment, Chang’an University, No. 126 Yanta Road, Xi’an 710054, China; 2 Key Laboratory of Subsurface Hydrology and Ecological Effects in Arid Region of the Ministry of Education, Chang’an University, No. 126 Yanta Road, Xi’an 710054, China

**Keywords:** M-MWCNTs, Mn(II) removal, kinetic model, isotherm model, PSO-BP model

## Abstract

Multiwalled carbon nanotubes (MWCNTs) were modified by oxidation and acidification with concentrated HNO_3_ and H_2_SO_4_, and the modified multiwalled carbon nanotubes (M-MWCNTs) and raw MWCNTs were characterized by several analytical techniques. Then the demanganization effects of MWCNTs and M-MWCNTs were well investigated and elucidated. The experimental data demonstrated that the adsorption efficiency of Mn(II) could be greatly promoted by M-MWCNTs from about 20% to 75%, and the optimal adsorption time was 6 h and the optimal pH was 6. The results of the kinetic model studies showed that Mn(II) removal by M-MWCNTs followed the pseudo-second-order model. Isothermal studies were conducted and the results demonstrated that the experimental data fitted well with the three models. The reliability of the experimental results was well verified by PSO–BP simulation, and the present conclusion could be used as a condition for further simulation. The research results provide a potential technology for promoting the removal of manganese from wastewater; at the same time, the application of various mathematical models also provides more scientific ideas for the research of the mechanism of adsorption of heavy metals by nanomaterials.

## 1. Introduction

Manganese, a heavy metal, is abundant in nature and also plays a significant role in many important industries [1,2]. However, due to factors such as artificial mining of mineral resources, illegal discharge of pollutants from factories, and dissolution of manganese minerals in aquifers caused by changes in ecological environment, the problem of pollution has become increasingly prominent [3,4,5]. In recent years, the concentration of manganese in underground wells in many countries has far exceeded the standards of the World Health Organization [6,7,8,9]. Overexposure to manganese can cause a variety of negative health effects for humans [10,11], and high manganese content in plants will lead to crop necrosis and cotton wrinkling, thus affecting the food and textile industry [12]. Studies have shown that in manganese-polluted areas, the soil and groundwater are usually containing acidic organic matter, so manganese mostly exists in the form of divalent ions [13,14,15].

There have been some studies on methods for the removal of manganese from a solution, such as the oxidation precipitation method [16], the ion exchange method [17], the reverse osmosis method [18] and the adsorption method, which is the more commonly used removal method [19,20,21]. Recently, with the development of nanomaterials, carbon nanotubes have been reported as the new adsorbents for the removal of heavy metal and organic pollution, such as chlorobenzenes, herbicides, heavy metal ions (Pb^2+^ and Ca^2+^), and inorganic nonmetallic ions, including F^−^ [22,23].

Due to lower synthesis and purification costs, and easy application to water treatment, multi-walled carbon nanotubes (MWCNTs) are more widely used than single-walled carbon nanotubes (SWCNTs) [24]. To improve the removal of heavy metals by carbon nanotubes, the original MWCNTs usually need to be modified. The ordinary modification method is to use a strong oxidant to oxidize the MWCNTs under reflux or ultrasonic conditions [25,26]. This oxidation endows carbon nanotubes with rich, oxygen-containing groups and exposes the adsorption site [17,27]. Meanwhile, in order to solve complex nonlinear problems in practice, some neural network models, such as artificial neural networks (ANN) and backpropagation-based training optimization neural networks (BPNN), have been gradually applied in the field of pollutant removal [28,29,30].

It has been reported that MWCNTs can adsorb manganese [31], but the removal efficiency, adsorption mechanism, and related experimental verification work have not been clearly carried out. Therefore, the objectives of this work are as follows: (1) to modify raw MWCNTs and to characterize the MWCNTs and M-MWCNTs by SEM, XPS, FT-IR, etc.; (2) to study the effects of demanganization on MWCNTs and M-MWCNTs with varying pH, contact time, and temperature; (3) to describe the characteristics of the Mn(II) removal by the MWCNTs and M-MWCNTs with an adsorption kinetic model and an isotherm model; and (4) to simulate the adsorption process by using PSO-BP modeling.

## 2. Results and Discussion

### 2.1. Characterization of MWCNTs and M-MWCNTs

#### 2.1.1. FT-IR

**Figure 1:**
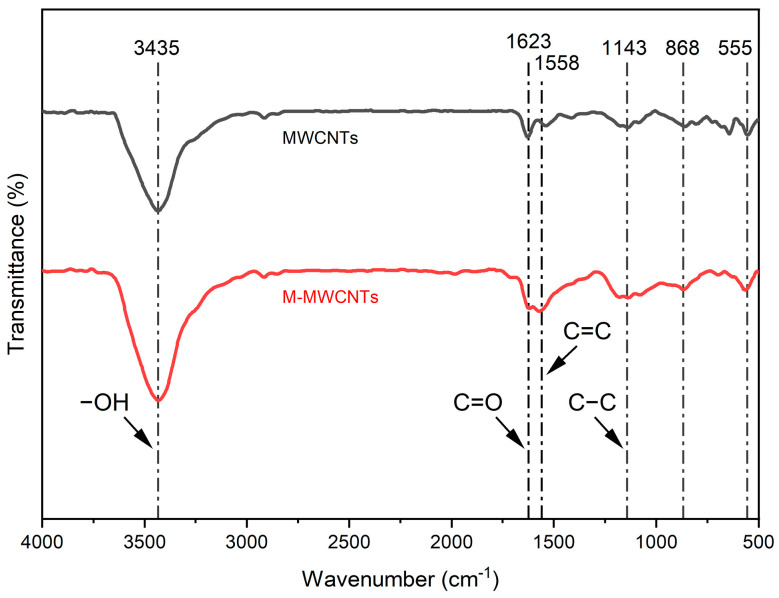
FTIR spectra of MWCNTs and M-MWCNTs.

The most important use of infrared spectroscopy (FTIR) is the structural analysis of organic compounds [32]. In this research, FT-IR was used to verify the structural analysis of MWCNTs and M-MWCNTs, and M-MWCNTs were modified by oxidation and acidification. The FTIR spectra of Figure 1 illustrated that there were functional groups, −OH groups (3200~3600 cm^−1^), −C=O− groups (1600 cm^−1^), and −C−C− groups (1150 cm^−1^), on the external and internal surface of MWCNTs and M-MWCNTs [33]. The transmittance (%) of the −OH groups and −C=O− groups in M-MWCNTs were stronger than in raw MWCNTs; the modification increased the active sites on the surface and further altered the surface polarity and charges. It is reported that the stretching vibration absorption peak of −C=O− usually appears in 1755–1670 cm^−1^. In this study, an absorption peak of −C=O− is observed at 1623 cm^−1^. This phenomenon has also shown up in the work of other researchers [34], possibly because the conjugate effect of carbon nanotubes makes the absorption of −C=O move to the shortwave direction [35].

#### 2.1.2. XPS

**Figure 2:**
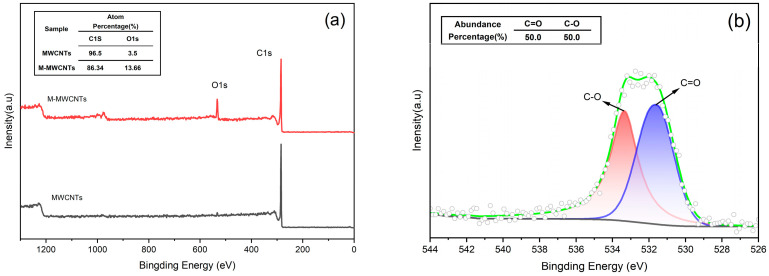
XPS spectra. (**a**) XPS of MWCNTs and M-MWCNTs (**b**) O1s XPS of M-MWCNTs.

XPS can be used to analyze elements present on the surface of the sample and bonding species [36]. The XPS wide-scan spectrum (Figure 2a) shows that the elements present on the surface of MWCNTs and M-MWCNTs were mainly C and O. New active sites are provided, as evidenced by the increased oxygen content of M-MWCNTs. This indicates that the adsorption capacity of M-MWCNTs for heavy metals will be enhanced [37]. The increase in O content (from 3.5% to 13.66%) showed that the modification was successful. Figure 2b shows the fitted XPS spectra of the O1s of M-MWCNTs compared to the reference XPS of MWCNTs and M-MWCNTs; the experimental data showed that −C=O− groups (530.7 eV) and −C−O− groups (533.6 eV) were on the surface of the M-MWCNTs.

#### 2.1.3. SEM

**Figure 3:**
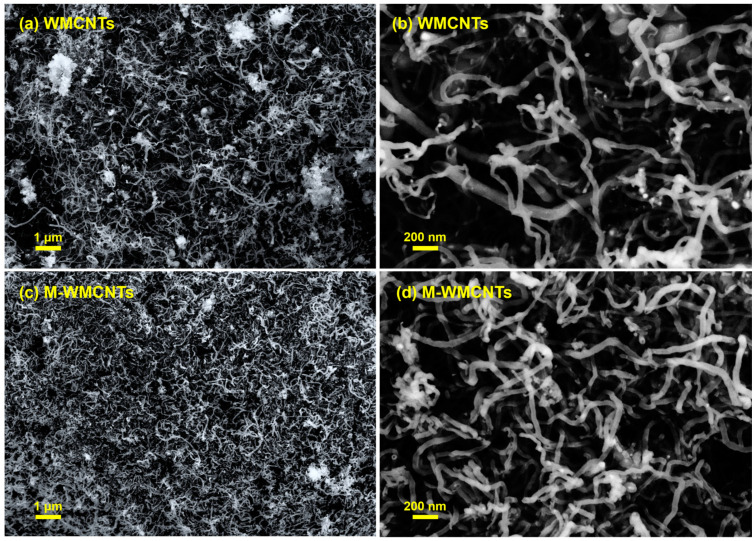
SEM of MWCNTs (**a**,**b**) and M-MWCNTs (**c**,**d**).

The morphology investigation of MWCNTs and M-MWCNTs was performed using SEM (Figure 3). Figure 3b shows that the MWCNTs and M-MWCNTs were about 20 nm in diameter, M-MWCNTs were shorter than MWCNTs, and reunions were more likely to occur in M-MWCNTs particles, a phenomenon that was consistent with the Zeta potential results.

#### 2.1.4. Zeta Potentials

**Figure 4:**
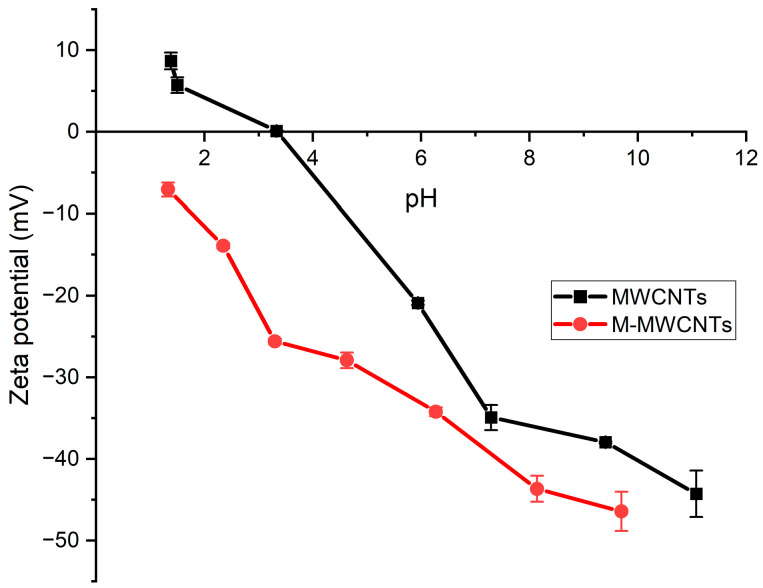
Zeta potentials of MWCNTs and M-MWCNTs.

The zeta potentials were measured as a function of pH to determine the pH_PZC_ of MWCNTs and M-MWCNTs [38]. The results (Figure 4) showed that the zeta potentials of MWCNTs and M-MWCNTs decreased with the increasing pH, whereas the zeta potentials of M-MWCNTs became more negative after the treatment. The zeta potentials of M-MWCNTs were all less than 0. It can be speculated that the reason for this phenomenon was the influence of certain groups (−COOH, −OH) [31]. Surface negativity is favorable for the adsorption of heavy metal ions from the solution by the adsorbent [39].

#### 2.1.5. Size

**Figure 5:**
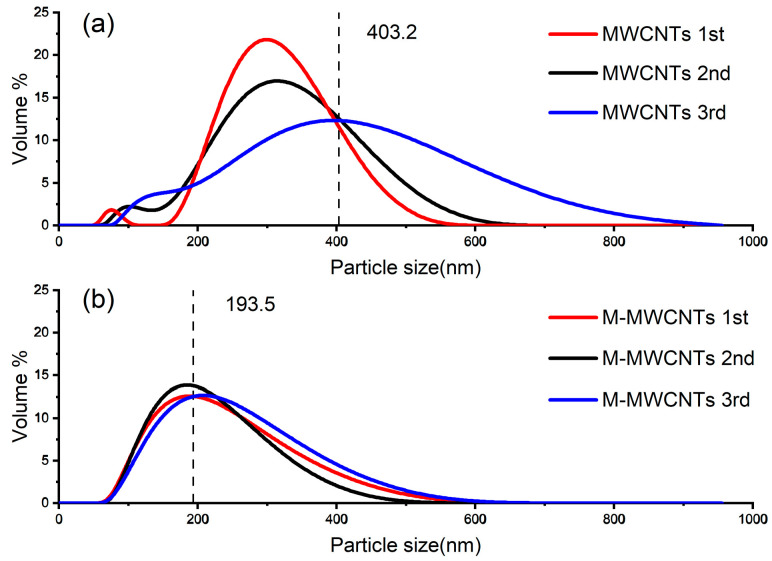
Results of nano-particle sizing (**a**) MWCNTs (**b**) M-MWCNTs.

Figure 5 shows the size distribution of MWCNTs and M-MWCNTs at pH 6.2. In general, the dynamic light-scattering results from MWCNTs represent agglomerations rather than individual nanomaterials [40]. In the measurement process, the agglomeration of the original MWCNTs leads to a wider particle size distribution and an increase in the mean value (Figure 5a). This phenomenon becomes more obvious over time (Figure 5a blue line). The average particle size of M-MWCNTs treated with mixed acid decreased, and the result of the three measurements was close to 193.5, indicating that the stability of carbon nanotubes in an aqueous solution was significantly improved. It is remarkable that, compared with the raw M-MWCNTs, the particle size of the M-MWCNTs with adsorbed Mn(II) was increased significantly; the average hydrated particle size increased from 193.5 nm to 320 nm.

### 2.2. Effect of Contact Time

**Figure 6:**
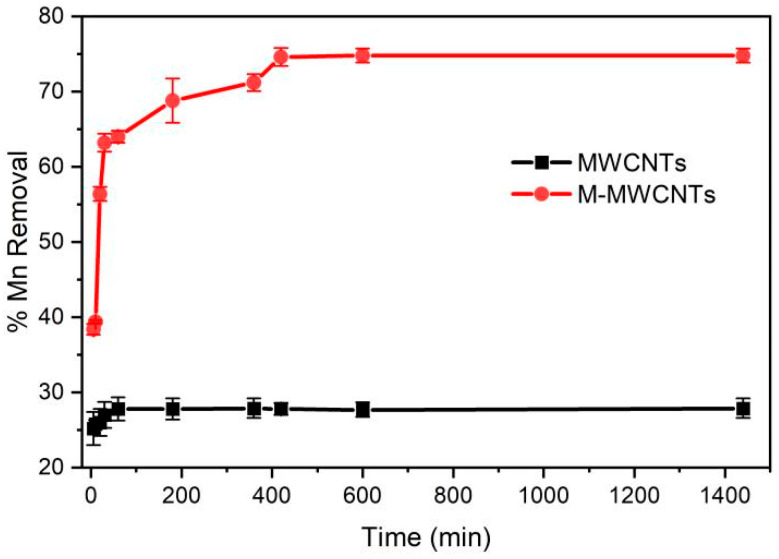
Contact time for Mn(II) adsorption by MWCNTs and M-MWCNTs.

The effect of contact time was studied under the following conditions: agitation speed, 180 rpm; adsorbent, 20 mg; initial concentration, 5 mg/L; pH, 5.6; temperature, 25 °C; and the mass ratio of adsorbent in solution was 1 g/L. The results are presented in Figure 6. It can be observed that the adsorption of Mn(II) onto the MWCNTs reached equilibrium rapidly within 1 h, but the removal efficiency of Mn(II) was low (27.9%). The adsorption of Mn(II) by M-MWCNTs increased rapidly in the first 30 min, then increased at a slower rate and reached equilibrium at 6 h. This result is corroborated in the literature [31].

### 2.3. Effect of pH

**Figure 7:**
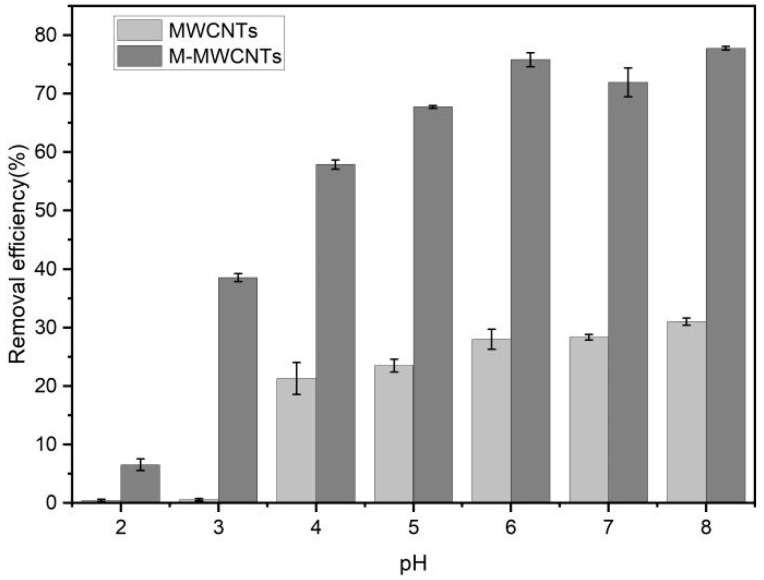
pH and Mn(II) adsorption by MWCNTs and M-MWCNTs.

The effect of pH on the demanganization by MWCNTs and M-MWCNTs is shown in Figure 7. The pH value varied from 2 to 9, contact time was 10 h, mass of adsorbent was 20 mg, concentration of Mn(II) was 5 mg/L, agitation speed was 180 rpm, the mass ratio of adsorbent in solution was 1 g/L, and the experimental temperature was 25 °C. The results indicated that the removal efficiency of Mn(II) increased. When the pH value is higher than 9, Mn(II) should be formed and Mn(OH)_2_ can even be precipitated [41]. In addition, under acidic conditions, H^+^ ions compete for the active sites with Mn(II) ions. As the pH increases, the concentration of H^+^ ions decreases, leaving more adsorption sites for Mn(II) ions. As the pH continues to increase, the Mn(II) is hydrolyzed, forming Mn(OH)^+^, Mn(OH)_2_, Mn_2_(OH)^+^_3_, and Mn(OH)^−^_4_. When pH > 8, the Mn(II) begins to form a precipitate [42].

When pH < 3, the adsorption rate of Mn(II) onto raw MWCNTs is close to 0. In the strong acid solution, the adsorption effect of raw MWCNTs on Mn(II) was inhibited, mainly due to a competitive effect between H^+^ and Mn^2+^ at the active site. The zeta potential results also support this conclusion; experimental zeta potential results showed that the zeta potential of the raw MWCNTs was positive in a solution of pH < 3 [43].

### 2.4. Kinetic Modeling

**Figure 8:**
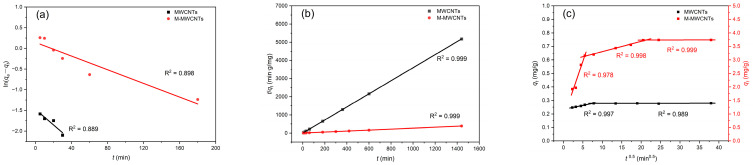
Adsorption kinetics. (**a**) Pseudo-first order, (**b**) pseudo-second order, and (**c**) intraparticle diffusion for Mn(II) adsorption by MWCNTs and M-MWCNTs.

The results of the three kinetic models are shown in Figure 8a,b, and the parameters are shown in Table 1. The results revealed that the adsorption of Mn(II) onto MWCNTs and M-MWCNTs followed second-order kinetics, which suggests that the adsorption process before both is chemical adsorption. The fitting line of the Weber–Morris model (Figure 8c) showed that the adsorption of Mn(II) onto MWCNTs and M-MWCNTs could be described in two or three stages, indicating that the Mn(II) adsorption process is controlled by some diffusion mechanisms, probably including an intra-particle diffusion mechanism, bulk diffusion, and film diffusion mechanisms.

### 2.5. Isotherm Modeling

**Figure 9:**
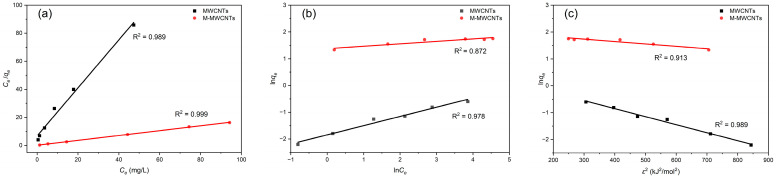
Isotherm models for Mn(II) adsorption by MWCNTs and M-MWCNTs. (**a**) Langmuir, (**b**) Freundlich, and (**c**) D–R.

The results of experimental data fitting to three adsorption isotherm models for Mn(II) are presented in Table 2 and Figure 9.

The adsorption of Mn(II) by MWCNTs simultaneously conformed to three models; all the values of *R*^2^ were >0.97 in the three isotherms. This indicated that the adsorption of Mn(II) by MWCNTs was complex and contained a variety of mechanisms under experimental conditions; the adsorption process had both monolayer and inhomogeneous surface chemisorption. As for M-MWCNTs, the *R*^2^ of the Langmuir model was close to 1, which is better than others. This indicated that the adsorption process of Mn(II) by M-MWCNTs was monolayer adsorption. As the Mn(II) ions attached to the site of M-MWCNTs (−COOH, −OH), no further adsorption occurred at that site [44]. Consequently, the adsorption capacity reached the maximum when the monolayer adsorption of Mn(II) ions was completely formed on the surface. The value of theoretical adsorption capacity calculated for M-MWCNTs is approximately 10 times higher than that of MWCNTs. The values of *R_L_* were from 0 to 1 for the studied concentration range, which means the adsorption of Mn(II) by M-MWCNTs is considered favorable.

Notably, the MWCNT and M-MWCNT results followed the D–R model well (*R*^2^ = 0.989 and 0.913, respectively). The value of *E_S_* indicated that the MWCNT adsorption process was mainly ion-exchange adsorption, while that of M-MWCNTs was mainly chemical adsorption [45].

### 2.6. PSO–BP Modeling

In this study, 35 and 39 sets of experiments were designed for MWCNTs and M-MWCNTs, respectively, and each set of experiments was conducted thrice. All data (35 × 3, 39 × 3, Table A1) were used for model fitting in order to reveal the inherent mechanisms in the process [46]. Imported data were normalized, randomly shuffled, and divided into three groups for crossover verification (70% for training, 15% testing, and 15% validation).

**Figure 10:**
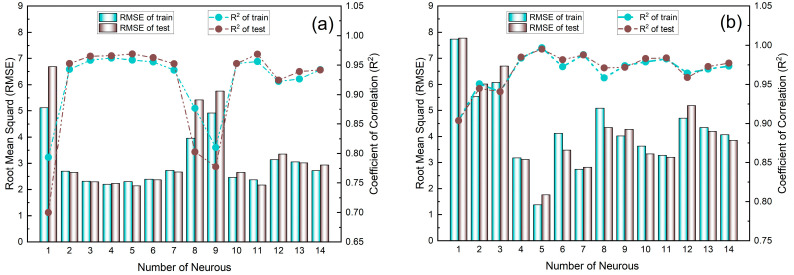
Variation in RMSE and R2 with number of neurons in hidden layer for Model A (**a**) and Model B (**b**).

**Figure A1:**
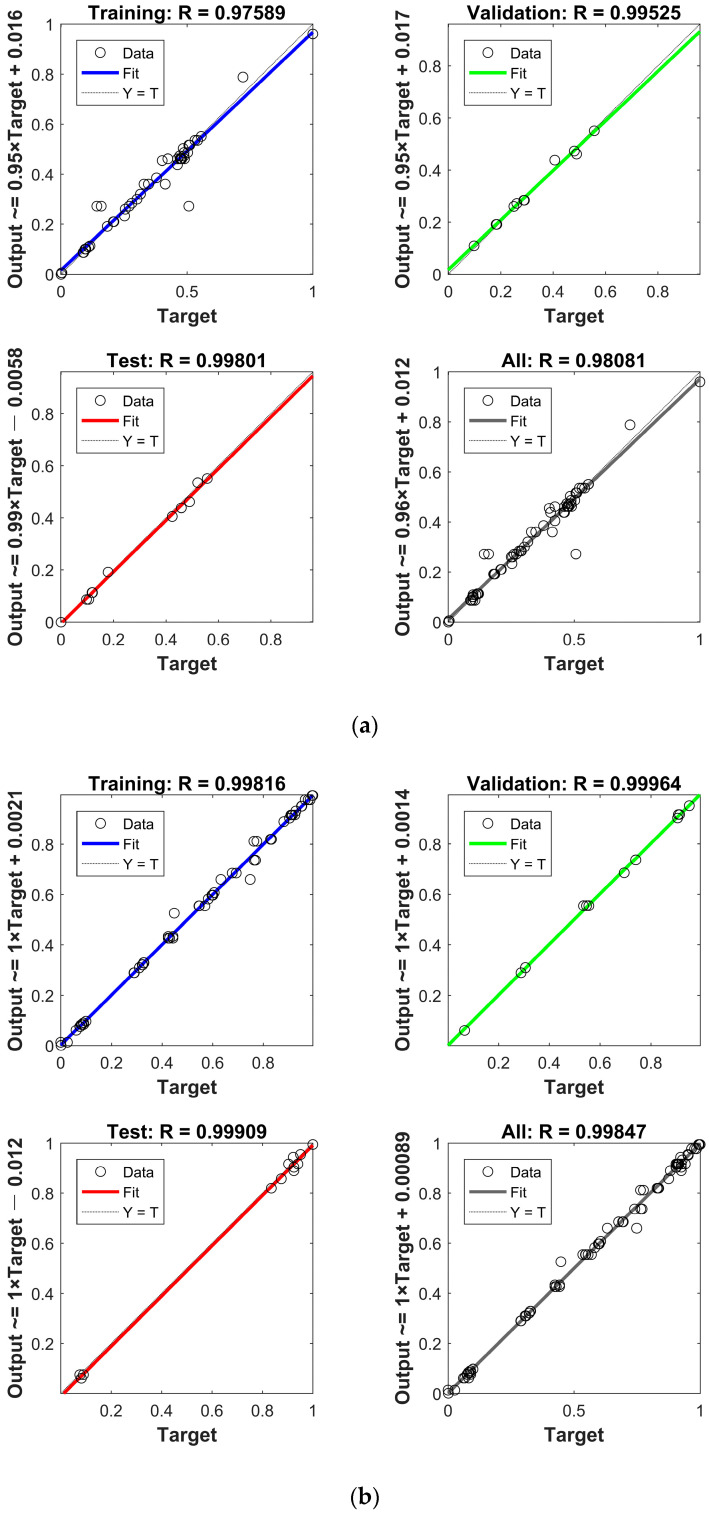
(**a**) Performances of model A on training, testing, validation, and combined data sets. (**b**) Performances of model B on training, testing, validation, and combined data sets.

In Figure 10, two models were developed for adsorption of MWCNTs (Model A) and M-MWCNTs (Model B), to choose the optimal network structure, 1–14 neurons were applied in the hidden layer, RMSE and R2 were used to evaluate the effectiveness of PSO-BP, and in the selected range, Figure 10 shows that the calculated results of the model were consistent with the experimental data, and for the value of R2, 11 of the 14 neurons had values around 0.95 in Figure 10a, and 12 of the 14 neurons of values ranged from 0.95 to 1.00 in Figure 10b. For the value of RMSE, in Figure 10a, 11 values were between 2.5–3, and in Figure 10b, the 10 values ranged from 3–5. The parameters of the PSO–BP model are listed in Table 3. With this fixed parameter, the optimal weights and biases obtained from the two PSO–BP models are shown in Table A2. Figure A1 shows the comparison between the input and output data of two models. The values of R for the training, testing, validation, and all data of both models were better than 0.97, which demonstrated that the predicted data agreed well with the experimental data using the PSO–BP model. Most of the data were distributed on the line of Y = T, indicating good compatibility of the experimental data with the PSO–BP-forecasted data. To further validate the performance of the model predictions, the four non-linear statistics of the model were evaluated. The results of the evaluation (Table 4) demonstrated that the predictions of both models were statistically significant.

## 3. Materials and Methods

### 3.1. Materials

The raw MWCNTs were purchased from COCC (Chengdu Institute of Organic Chemistry, Chinese Academy of Science). Using carbon gas as the carbon source, this material was produced via the CVD (catalytic vapor decomposition) method; its outer diameter was between 5 and 15 nm, the surface area was in the range of 220–300 m^2^/g, and the purity of the material was above 95%, with low metal impurity content and high electrical conductivity.

Mn(II) stock solution (1000 mg/L) was prepared by dissolving 3.6010 g MnCl_2_•4H_2_O (GR) in 1000 mL of deionized water. All the manganese-containing solutions in later experiments were diluted from the stock solution. The concentration of Mn(II) was determined by atomic absorption spectrometry (WFX120).

### 3.2. Preparation of M-MWCNTs

**Figure 11:**
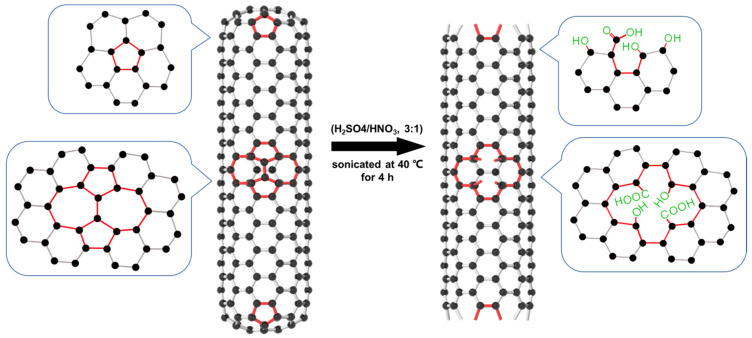
Schematic diagram of the preparation of M-MWCNTs.

A total of 5 g of raw MWCNTs was added to a 120 mL mixture of HNO_3_ (68%) and H_2_SO_4_ (98%) (*v*/*v* 1:3). The mixture was sonicated at 40 °C for 4 h and stirred at room temperature for 24 h, so that the MWCNTs were fully in contact with the oxidant and were oxidized. The resulting MWCNTs were separated from the solution using a 0.22 μm membrane filter, rinsed thoroughly with deionized water to remove excess acid, and then rinsed with anhydrous ethanol to accelerate drying. The obtained sample was dried at 65 °C for 24 h. Figure 11 shows the preparation of M-MWCNTs.

### 3.3. Characterization Analysis Methods

The FT-IR spectra of the dried samples were determined by an IRTracer 100 FTIR spectrometer (Shimadzu, Japan), using the potassium bromide powder method. The range of scanning was 4000–350 cm^−1^ and the resolution was set to 4 cm^−1^.

XPS (X-ray photoelectron spectroscopy) was performed to investigate the creation of ionic networks between MWCNTs and M-MWCNTs on ESCALAB Xi+. The main parameters were spatial resolution (1 μm) and energy resolution (0.48 eV). The sample needed to be pressed before testing.

SEM (scanning electron microscopy, Zeiss Ultra Plus) was used to detect the surface morphology of the MWCNTs and M-MWCNTs [47] and the micro area constitution of materials was analyzed using Oxford X-Max 50 mm Energy Disperse Spectroscopy (EDS).

A Malvern Zetasizer Ultra was used to measure the particle sizes and zeta potentials of MWCNTs and M-MWCNTs. The samples were dispersed by ultrasonication for 20 min before measurement.

### 3.4. Batch Experiments

Adsorption experiments. A total of 20 mg MWCNTs and 20 mg M-MWCNTs were separately mixed with 20 mL of Mn(II) solution in a polypropylene centrifuge tube of 50 mL. The initial Mn(II) concentration was 5, 10, 20, 50, 80, and 100 mg/L, while the initial pH value was from 5.6 to 5.2. The mixture was shaken for 10 h and 25 °C at 180 rpm, then filtered using a 0.22 μm membrane filter.

Adsorption thermodynamics experiments. The temperature was set at 25, 35, 45, and 55 °C, and the contact time for adsorption kinetics was controlled from 5 min to 24 h. To study the effect of pH, the pH value was adjusted using sodium hydroxide solution (0.01~0.1 mol/L) and hydrochloric acid solution (0.01~0.1 mol/L).

### 3.5. Theory

#### 3.5.1. Kinetic Modeling

The pseudo-first-order model, pseudo-second-order model, and Weber–Morris model were the kinetic models used to describe the mechanism of Mn(II) adsorption onto MWCNTs and M-MWCNTs. The pseudo-first-order kinetic model can be used to fit the materials with fewer active sites, higher initial adsorption concentration and the adsorption process in the early stage, while the pseudo-second-order kinetic model is just the opposite [48]. Pseudo-first-order and pseudo-second-order dynamics models belong to empirical models. They are simple to calculate, but they fail to explain the adsorption process in terms of mechanism. A Weber–Morris dynamics model has been put to use in the adsorption of metal ions by many scholars [49]. It can be used to analyze the rate-controlling steps of the adsorption reaction, which can guide the adsorption process.

The pseudo-first-order model, which is widely used in kinetic adsorption simulation, is based on the assumption that adsorption is controlled by diffusion steps [50]. Equation (1) describes this model, where $q_{e}$ is the equilibrium absorption capacity, $q_{t}$ is the absorption capacity at time *t*, and $k_{f}$ is the rate constant. Equation (2) is the integrated form.

(1) $$\left(dq_{t})/dt=k_{f}(q_{e}-q_{t}\right)$$

(2) $$\log\left(q_{e}-q_{t}\right)=\log\left(q_{e}\right)-k_{f}t/2.303$$

The pseudo-second-order kinetic model assumes that the adsorption rate is determined by the square value of the number of vacant adsorption spots on the adsorbent surface, and the adsorption process is controlled by the chemisorption mechanism, which involves electron sharing or electron transfer between the adsorbent and the adsorbate [51]. Equation (3) describes this model, in which $q_{e}$ is the equilibrium absorption capacity, $q_{t}$ is the absorption capacity at time *t*, and $k_{s}$ is the constant. Equations (4) and (5) are the integrated forms.

(3) $$(dq_{t})/dt=k_{s}{\left(q_{e}-q_{t}\right)}^{2}$$

(4) $$1/(q_{e}-q_{t})=1/q_{e}+k_{s}t$$

(5) $$t/q_{t}=1/k_{s}{q_{e}}^{2}+t/q_{e}$$

The Weber–Morris dynamics model is a particle diffusion model (Equation (6)) [52,53]. The model is the most suitable for describing the dynamics of the material diffusion process inside the particle, but it is often not suitable for describing the diffusion process outside the particle and inside the liquid film.

(6) $$q_{t}=k_{w}t^{1/2}$$

In Equation (6), $q_{t}$ is the absorption capacity and *k_w_* is the rate constant.

#### 3.5.2. Isotherm Modeling

The adsorption isotherm model is a mathematical model that expresses the relationship between the adsorption capacity and the solution concentration under the condition of fixed temperature. Different types of mathematical expression have been proposed and each has its own scope of application; commonly used expressions are the Langmuir, Freundlich, and Dubinin–Radushkevich isotherm models.

The Langmuir model assumes that the appearance of the adsorbent is uniform and there is no interaction between adsorbents. The adsorption is monolayer adsorption, and the adsorption occurs only on the outer surface of the adsorbent. The Langmuir isothermal adsorption model was the first model to vividly describe the adsorption mechanism [54]. Equations (7) and (8) describe the linearized form [55], where $q_{e}$ is the equilibrium absorption capacity, $C_{e}$ is the equilibrium concentration, $q_{m}$ is the maximum equilibrium absorption capacity, and *b* is the Langmuir rate constant.

(7) $$q_{e}=\frac{q_{m}bC_{e}}{1+bC_{e}}$$

(8) $$\frac{C_{e}}{q_{e}}=\frac{1}{q_{m}b}+\frac{1}{q_{m}}C_{e}$$

Equation (9) is the essential expression for the Langmuir model, and it is easy to estimate the characteristics of adsorption.

(9) $$R_{L}=\frac{1}{1+bC_{0}}$$

$R_{L}$ is a dimensionless constant and $C_{0}$ is the concentration of the Mn(II) solution. $R_{L}$ values between 0 and 1 indicate favorable adsorption [56].

The Freundlich model can be applied to monolayer adsorption and heterogeneous surface adsorption. Not only can the Freundlich adsorption equation describe the adsorption mechanism of an uneven surface, it is also more suitable for adsorption at low concentrations. It can explain the experimental results over a wider range of concentration. The Freundlich isotherm (Equations (10) and (11)) [57] can be expressed as:

(10) $$q_{e}=K_{f}{C_{e}}^{1/n}$$

(11) $$lnq_{e}=lnK_{f}+\frac{1}{n}\ln C_{e}$$

where $q_{e}$ is the equilibrium absorption capacity, $C_{e}$ is the equilibrium concentration, $K_{f}$ is the Freundlich constant, and the n value ranges from 1 to 10.

In order to describe the relative pressure–adsorption capacity characteristics of microporous filling, Dubinin and Radushkevich proposed a method, based on the Polanyi adsorption potential theory, to calculate the adsorption characteristics based on the adsorption isotherm in the low-pressure region. The Dubinin–Radushkevich (D-R) isotherm is shown in Equation (12) [58]:

(12) $$lnq_{e}=lnq_{m}-K\varepsilon^{2}$$

(13) $$\varepsilon =RTln(1+1/C_{e})$$

where $q_{e}$ is the absorption capacity, $q_{m}$ is the theoretical adsorption capacity, $C_{e}$ is the equilibrium concentration, and K is the D-R constant.

In order to describe the calculation results of this model more easily, the relationship between the free adsorption energy, *E_s_*, and the constant, *K*, is given as Equation (14). The value of *Es* determines the mechanism of isothermal adsorption.

(14) $$E_{s}={\left(2K\right)}^{-0.5}$$

If the value of *E_s_* is between approximately 8 and 16 kJ/mol, it shows that the main process of adsorption is ion exchange, whereas if the value *E_s_* is more than 18 kJ/mol, it means the adsorption is chemisorptive in nature.

#### 3.5.3. PSO-BP Modeling

Since Warren McCulloch and Walter Pitts introduced the concept of artificial neural networks (ANN) in 1943, ANN have evolved rapidly and have been successfully applied in many fields [59]. Backpropagation-based training-optimization neural networks (BPNN) are the most extensively utilized neural networks in practice and are capable of solving complex nonlinear problems. BPNN also have a wide range of applications in the field of pollutant removal [28,29,30]. BPNN require constant exploration of different combinations of weights and biases to obtain ideal results. Particle swarm optimization (PSO) algorithms are widely used for this work. PSO is a stochastic search algorithm that simulates the predatory behavior of birds. A set of optimal solutions was obtained from the PSO algorithm by tracking the constantly moving particles.

In this study, a BPNN model optimized by PSO (PSO–BP) was used to predict the adsorption of Mn onto two carbon materials. This process was implemented with Matlab2016a software. A standard BPNN network with one input layer, one hidden layer, and one output layer was created by calling on the software’s own “newff” function. The backpropagation algorithm, which was applied to determine the most favorable network structure, selected Levenberg–Marquardt (trainlm) with 1000 iterations. The input layer contains four neurons (pH, manganese concentration, time, and temperature). The output is the percentage of Mn(II) removed. Owing to the small amount of data, the network uses just one hidden layer to avoid overfitting [60] and uses a tangent sigmoid function (tansig) as the activation function, whereas the output layer uses a linear function (purlin) as the transfer function. PSO uses the weights and MSE of the network as the particle and fitness functions, respectively, to learn and iterate. After each iteration, the new particles are transformed into the weights of the neural network; the network computes a new MSE and continues to iterate until the result is ideal or the number of iterations reaches a maximum.

The results of the model calculations are evaluated by the following equations (Equations (15)–(18)):

(15) $$R^{2}=1-\frac{\sum_{i=1}^{n}{\left(y_{prd,i}-y_{exp,i}\right)}^{2}}{\sum_{i=1}^{n}{\left(y_{prd,i}-y_{m}\right)}^{2}}$$

(16) $$RMSE=\sqrt{\frac{1}{n}\sum_{i=1}^{n}{\left(y_{prd,i}-y_{exp,i}\right)}^{2}}$$

(17) $$MAE=\frac{1}{n}\sum_{i=1}^{n}\left|y_{prd,i}-y_{exp,i}\right|$$

(18) $$MAPE=\frac{100\%}{n}\sum_{i=1}^{n}\left|\frac{y_{prd,i}-y_{exp,i}}{y_{exp,i}}\right|$$

where $y_{prd,i}$ is the value calculated using the PSO–BP model, $y_{exp,i}$ is the experimental value, n is the number of data, and $y_{m}$ is the average of the experimental value.

## 4. Conclusions

Compared with previous studies, the Mn(II)-removal performance by M-MWCNTs was improved [31]. Through the comparative analysis of several analytical techniques results before and after modification, as well as the fitting of thermodynamic and kinetic adsorption models, it was concluded that the increased number of adsorption sites (carboxyl groups and hydroxyl groups) after modification was the key to improving the removal rate. Additionally, the simulation verification of the experimental results by PSO–BP model also provided a scientific guarantee for the reliability of the entire research work.

## Figures and Tables

**Table 1:** Pseudo-first-order, pseudo-second-order and intra-particle diffusion model constants for Mn(II) adsorption by MWCNTs and M-MWCNTs.

Model	Parameter	MWCNTs	M-MWCNTs
	*q_e.experiment_*	0.278	3.74
Pseudo-first-order model	*q_e.calculate_* (mg/g)	0.229	1.16
	*k_f_* (1/min)	0.0433	0.019
	*R* ^2^	0.889	0.898
Pseudo-second-order model	*q_e.calculate_* (mg/g)	0.279	3.76
	*k_s_* (1/min)	4.12	0.030
	*R* ^2^	0.999	0.999
Weber–Morris	*k_w_*	/	/
	*C*	/	/
	*R* ^2^	/	/

**Table 2:** Isotherm parameters and determination coefficients for Mn(II) adsorption by MWCNTs and M-MWCNTs.

Model	Parameter	MWCNTs	M-MWCNTs
Langmuir isotherm	*q_max_*_1_ (mg/g)	0.585	5.78
	*b* (L/mg)	0.249	1.19
	*R_L_* (L/mg)	0.801	0.456
	*R* ^2^	0.989	0.999
Freundlich isotherm	*K_f_* (mg/g (L/mg)^1/n^)	0.159	3.96
	1/*n*	0.344	0.009
	*R* ^2^	0.978	0.872
Dubinin–Radushkevich	*q_max_*_2_ (mg/g)	1.4083	7.43
	*β*	0.0030	0.0009
	*E_S_*	12.95	23.64
	*R* ^2^	0.989	0.913

**Table 3:** The parameters of the optimum PSO–BP model.

Parameter	Model A: MWCNTs	Model B: M-MWCNTs
Number of samples	117	105
Number of hidden layers	1	1
Hidden nodes	4	5
PSO swarm size	50	50
Cognitive component (c1)	1.5	1.49554
Social component (c2)	1.5	1.49554
Number of iterations	1000	1000

**Table 4:** Nonlinear statistical metrics for validating efficacy of model predictions.

Statistical Metric	Model A: MWCNTs	Model B: M-MWCNTs
*R* ^2^ * (train/test)*	0.962/0.966	0.997/0.995
*RMSE (train/test)*	2.20/2.26	1.38/1.76
*MAE (train/test)*	1.404/1.58	0.769/0.909
*MAPE*	0.074/0.067	0.0202/0.0223

## Data Availability

We can provide the data of this journal through the corresponding author’s email.

## Sample Availability

Samples of the compounds are available from the authors.

## Appendix A

**Table A1:** (a) Data used for model A. (b) Data used for model B.

**(a)**					
**No.**	**pH**	**C/mg/L**	**Time/h**	**T/K**	**Adsorption Rate %**
1	2.18	5	180	298	0.4
2	3.23	5	180	298	0.6
3	4.17	5	180	298	20.4
4	5.15	5	180	298	23.5
5	6.17	5	180	298	28.5
6	7.18	5	180	298	28.31
7	8.25	5	180	298	30.4
8	5.58	1	180	298	58
9	5.58	2	180	298	42
10	5.58	5	180	298	28.6
11	5.58	10	180	298	9.6
12	5.58	20	180	298	11
13	5.58	50	180	298	6.5
14	5.58	5	5	298	22.2
15	5.58	5	10	298	25.8
16	5.58	5	20	298	26.8
17	5.58	5	30	298	28
18	5.58	5	60	298	27
19	5.58	5	180	298	28.6
20	5.58	5	180	298	28.2
21	5.58	10	180	298	15.99
22	5.58	20	180	298	11.01
23	5.58	50	180	298	5.45
24	5.58	5	180	308	29.34
25	5.58	10	180	308	16.61
26	5.58	20	180	308	12.38
27	5.58	50	180	308	5.89
28	5.58	5	180	318	29.6
29	5.58	10	180	318	18.01
30	5.58	20	180	318	14.95
31	5.58	50	180	318	6.7
32	5.58	5	180	328	32.45
33	5.58	10	180	328	18.59
34	5.58	20	180	328	15.22
35	5.58	50	180	328	7.03
36	2.18	5	180	298	0.4
37	3.23	5	180	298	0.58
38	4.17	5	180	298	19.4
39	5.15	5	180	298	23.18
40	6.17	5	180	298	27.5
41	7.18	5	180	298	28.5
42	8.25	5	180	298	31
43	5.58	1	180	298	59
44	5.58	2	180	298	43
45	5.58	5	180	298	26.2
46	5.58	10	180	298	29.6
47	5.58	20	180	298	10.75
48	5.58	50	180	298	6
49	5.58	5	5	298	26.4
50	5.58	5	10	298	25.2
51	5.58	5	20	298	23.8
52	5.58	5	30	298	27.6
53	5.58	5	60	298	27
54	5.58	5	180	298	26.2
55	5.58	5	180	298	27.8
56	5.58	10	180	298	15.45
57	5.58	20	180	298	11.05
58	5.58	50	180	298	5.59
59	5.58	5	180	308	28.5
60	5.58	10	180	308	17.12
61	5.58	20	180	308	12.51
62	5.58	50	180	308	6.05
63	5.58	5	180	318	29.8
64	5.58	10	180	318	17.8
65	5.58	20	180	318	14.5
66	5.58	50	180	318	6.58
67	5.58	5	180	328	32.5
68	5.58	10	180	328	18.51
69	5.58	20	180	328	15.1
70	5.58	50	180	328	7.22
71	2.18	5	180	298	0.4
72	3.23	5	180	298	0.51
73	4.17	5	180	298	24.2
74	5.15	5	180	298	23.8
75	6.17	5	180	298	28.1
76	7.18	5	180	298	28.2
77	8.25	5	180	298	31.6
78	5.58	1	180	298	49
79	5.58	2	180	298	40.5
80	5.58	5	180	298	28.6
81	5.58	10	180	298	8.6
82	5.58	20	180	298	11.5
83	5.58	50	180	298	4
84	5.58	5	5	298	25.6
85	5.58	5	10	298	24.8
86	5.58	5	20	298	27
87	5.58	5	30	298	24.8
88	5.58	5	60	298	29.6
89	5.58	5	180	298	28.6
90	5.58	5	180	298	27.6
91	5.58	10	180	298	16.5
92	5.58	20	180	298	10.9
93	5.58	50	180	298	5.5
94	5.58	5	180	308	28.2
95	5.58	10	180	308	16.98
96	5.58	20	180	308	12.63
97	5.58	50	180	308	6.09
98	5.58	5	180	318	28.6
99	5.58	10	180	318	17.1
100	5.58	20	180	318	14
101	5.58	50	180	318	6.04
102	5.58	5	180	328	32.49
103	5.58	10	180	328	18.58
104	5.58	20	180	328	14.8
105	5.58	50	180	328	7.31
**(b)**					
**No.**	**pH**	**C/mg/L**	**Time/h**	**T/K**	**Adsorption Rate %**
1	2.36	5	1440	298	5.6
2	3.38	5	1440	298	37.8
3	4.32	5	1440	298	57
4	5.31	5	1440	298	74.3
5	6.3	5	1440	298	76.1
6	7.37	5	1440	298	75.6
7	8.32	5	1440	298	77.8
8	5.58	5	1440	298	75.8
9	5.58	10	1440	298	48.8
10	5.58	20	1440	298	27.6
11	5.58	50	1440	298	12.4
12	5.58	80	1440	298	11.8
13	5.58	100	1440	298	5.69
14	5.58	5	5	298	38.2
15	5.58	5	10	298	39.2
16	5.58	5	20	298	53.6
17	5.58	5	30	298	64
18	5.58	5	60	298	64.4
19	5.58	5	180	298	68.8
20	5.58	5	300	298	69.8
21	5.58	5	420	298	75.8
22	5.58	5	600	298	75.8
23	5.58	5	1440	298	75.8
24	5.58	5	1440	298	75.1
25	5.58	10	1440	298	47.1
26	5.58	20	1440	298	27.5
27	5.58	50	1440	298	11.4
28	5.58	5	1440	308	77.2
29	5.58	10	1440	308	49.8
30	5.58	20	1440	308	29
31	5.58	50	1440	308	12.4
32	5.58	5	1440	318	80.4
33	5.58	10	1440	318	50.9
34	5.58	20	1440	318	30
35	5.58	50	1440	318	12.4
36	5.58	5	1440	328	81
37	5.58	10	1440	328	52
38	5.58	20	1440	328	30.4
39	5.58	50	1440	328	13
40	2.36	5	1440	298	7.6
41	3.38	5	1440	298	38.6
42	4.32	5	1440	298	58.4
43	5.31	5	1440	298	73.8
44	6.3	5	1440	298	76.3
45	7.37	5	1440	298	75.4
46	8.32	5	1440	298	77.9
47	5.58	5	1440	298	77
48	5.58	10	1440	298	47
49	5.58	20	1440	298	27.4
50	5.58	50	1440	298	10.4
51	5.58	80	1440	298	10.2
52	5.58	100	1440	298	5.8
53	5.58	5	5	298	37.8
54	5.58	5	10	298	39.2
55	5.58	5	20	298	62.4
56	5.58	5	30	298	61.8
57	5.58	5	60	298	63.5
58	5.58	5	180	298	68.5
59	5.58	5	300	298	72
60	5.58	5	420	298	72.5
61	5.58	5	600	298	74.2
62	5.58	5	1440	298	74.2
63	5.58	5	1440	298	74.8
64	5.58	10	1440	298	47.8
65	5.58	20	1440	298	27.29
66	5.58	50	1440	298	11.41
67	5.58	5	1440	308	77.8
68	5.58	10	1440	308	49.8
69	5.58	20	1440	308	28.8
70	5.58	50	1440	308	11.9
71	5.58	5	1440	318	79.8
72	5.58	10	1440	318	51.2
73	5.58	20	1440	318	30.1
74	5.58	50	1440	318	12.5
75	5.58	5	1440	328	81.5
76	5.58	10	1440	328	51.1
77	5.58	20	1440	328	30.5
78	5.58	50	1440	328	13.1
79	2.36	5	1440	298	6.4
80	3.38	5	1440	298	39.2
81	4.32	5	1440	298	58.2
82	5.31	5	1440	298	74.5
83	6.3	5	1440	298	75.9
84	7.37	5	1440	298	75.2
85	8.32	5	1440	298	77.8
86	5.58	5	1440	298	74.6
87	5.58	10	1440	298	46.2
88	5.58	20	1440	298	28
89	5.58	50	1440	298	11.3
90	5.58	80	1440	298	10.6
91	5.58	100	1440	298	5.71
92	5.58	5	5	298	39.2
93	5.58	5	10	298	39.6
94	5.58	5	20	298	53
95	5.58	5	30	298	63.6
96	5.58	5	60	298	64.4
97	5.58	5	180	298	69
98	5.58	5	300	298	71.6
99	5.58	5	420	298	74.6
100	5.58	5	600	298	74.4
101	5.58	5	1440	298	74.5
102	5.58	5	1440	298	74.9
103	5.58	10	1440	298	47.1
104	5.58	20	1440	298	27.5
105	5.58	50	1440	298	11.39
106	5.58	5	1440	308	77.3
107	5.58	10	1440	308	49.8
108	5.58	20	1440	308	29.1
109	5.58	50	1440	308	11.7
110	5.58	5	1440	318	78.9
111	5.58	10	1440	318	51
112	5.58	20	1440	318	30
113	5.58	50	1440	318	12.3
114	5.58	5	1440	328	81.2
115	5.58	10	1440	328	51.6
116	5.58	20	1440	328	30.6
117	5.58	50	1440	328	13.1

**Table A2:** (a) Model A 4-4-1 topology—optimal weights and biases. (b) Model B 4-5-1 topology—optimal weights and biases.

**(a)**						
**Input (4)-Hidden Layer (4)**					**Hidden Layer (4)-Output (1)**	
**Weights**				**Bias**	**Weights**	**Bias**
−0.0021	−0.0998	0.0628	0.1912	−0.4088	−0.5668	−0.1690
−0.3308	−0.3013	0.0358	−0.0908	−0.3962	0.2999	
−0.1177	0.7891	0.1748	0.2355	−0.3528	−0.3022	
−0.2683	0.1825	0.0773	−0.4901	0.1691	−0.0722	
**(b)**						
**Input (4)-Hidden Layer (5)**					**Hidden Layer (5)-Output (1)**	
**Weights**				**Bias**	**Weights**	**Bias**
0.8852	−0.6705	−0.3712	0.0612	0.2464	0.1556	−0.3376
0.2801	0.1780	0.1084	−0.4348	0.3055	1	
−0.1308	0.4948	−0.0209	0.0709	−0.0502	0.3046	
−0.9012	−0.2605	−0.4838	−0.8644	0.2783	−0.4782	
0.6520	−1	−0.7253	0.1455	0.6593	−0.2843	

## References

[1] Clarke C., Upson S. (2017). A global portrait of the manganese industry—A socioeconomic perspective. NeuroToxicology.

[2] Singh T. (2020). A Review of Low Grade Manganese Ore Upgradation Processes. Miner. Process. Extr. Metall. Rev..

[3] Jandieri G. (2022). A generalized model for assessing and intensifying the recycling of metal-bearing industrial waste: A new approach to the resource policy of manganese industry in Georgia. Resour. Policy.

[4] Ghosh S., Mohanty S., Akcil A., Sukla L.B., Das A.P. (2016). A greener approach for resource recycling: Manganese bioleaching. Chemosphere.

[5] Marks A. (2017). Aquatic environmental risk assessment of manganese processing industries. Neurotoxicology.

[6] Vetrimurugan E., Brindha K., Elango L., Ndwandwe O.M. (2017). Human exposure risk to heavy metals through groundwater used for drinking in an intensively irrigated river delta. Appl. Water Sci..

[7] Therdkiattikul N., Ratpukdi T., Kidkhunthod P., Chanlek N., Siripattanakul-Ratpukdi S. (2020). Manganese-contaminated groundwater treatment by novel bacterial isolates: Kinetic study and mechanism analysis using synchrotron-based techniques. Sci. Rep..

[8] Masoud A.A., Koike K., Mashaly H.A., Gergis F. (2016). Spatio-temporal trends and change factors of groundwater quality in an arid area with peat rich aquifers: Emergence of water environmental problems in Tanta District, Egypt. J. Arid. Environ..

[9] Yu D., Zhou J., Zhang J., Sun Y., Zeng Y. (2021). Hydrogeochemistry and evolution of iron and manganese in groundwater in Kashgar, Xinjiang. Acta Sci. Circumst.

[10] Abubakar B., Jiun O.D., Ismail M., Imam M.U. (2019). Nutrigenomics and Antioxidants. Nutraceuticals and Natural Product Pharmaceuticals.

[11] Blanc P.D. (2017). The Early History of Manganese and the Recognition of its Neurotoxicity, 1837–1936. Neurotoxicology.

[12] Dey S., Tripathy B., Kumar M.S., Das A.P. (2023). Ecotoxicological consequences of manganese mining pollutants and their biological remediation. Environ. Chem. Ecotoxicol..

[13] Hou Q., Zhang Q., Huang G., Liu C., Zhang Y. (2020). Elevated manganese concentrations in shallow groundwater of various aquifers in a rapidly urbanized delta, south China. Sci. Total Environ..

[14] Champ D.R., Gulens J., Jackson R.E. (1979). Oxidation–reduction sequences in ground water flow systems. Can. J. Earth Sci..

[15] Dou Y., Li M., Liu Z., Fang R., Yan K. (2020). Simulation of magnesium chloride vertical transport in column experiments. Hum. Ecol. Risk Assess. Int. J..

[16] Szymoniak L., Claveau-Mallet D., Haddad M., Barbeau B. (2022). Improving remineralization and manganese-removal of soft waters using a mixed CaCO_3_/MgO contactor. J. Water Process Eng..

[17] Bikiaris D., Vassiliou A., Chrissafis K., Paraskevopoulos K.M., Jannakoudakis A., Docoslis A. (2008). Effect of acid treated multi-walled carbon nanotubes on the mechanical, permeability, thermal properties and thermo-oxidative stability of isotactic polypropylene. Polym. Degrad. Stab..

[18] Stein S., Sivan O., Yechieli Y., Kasher R. (2021). Redox condition of saline groundwater from coastal aquifers influences reverse osmosis desalination process. Water Res..

[19] Li M., Kuang S., Kang Y., Ma H., Dong J., Guo Z. (2022). Recent advances in application of iron-manganese oxide nanomaterials for removal of heavy metals in the aquatic environment. Sci. Total Environ..

[20] Nassar M.Y., El-Shahat M.F., Osman A., Sobeih M.M., Zaid M.A. (2021). Adsorptive Removal of Manganese Ions from Polluted Aqueous Media by Glauconite Clay-Functionalized Chitosan Nanocomposites. J. Inorg. Organomet. Polym. Mater..

[21] Vasile E., Covaliu C.I., Stoian O., Paraschiv G., Ciric A. (2020). Manganese Ions Removal from Industrial Wastewater. Rev. De Chim.-Buchar. Orig. Ed..

[22] Dehghani M.H., Haghighat G.A., Yetilmezsoy K., McKay G., Heibati B., Tyagi I., Agarwal S., Gupta V.K. (2016). Adsorptive removal of fluoride from aqueous solution using single- and multi-walled carbon nanotubes. J. Mol. Liq..

[23] Rodríguez C., Leiva E. (2020). Enhanced Heavy Metal Removal from Acid Mine Drainage Wastewater Using Double-Oxidized Multiwalled Carbon Nanotubes. Molecules.

[24] Mishra S., Sundaram B. (2022). Efficacy and Challenges of Carbon Nanotube in Wastewater and Water Treatment. Environ. Nanotechnol. Monit. Manage..

[25] Aslam M., Kuo H.W., Den W., Usman M., Ashraf H. (2021). Functionalized Carbon Nanotubes (CNTs) for Water and Wastewater Treatment: Preparation to Application. Sustainability.

[26] Ihsanullah, Abbas A., Al-Amer A.M., Laoui T., Al-Marri M.J., Nasser M.S., Khraisheh M., Atieh M.A. (2016). Heavy metal removal from aqueous solution by advanced carbon nanotubes: Critical review of adsorption applications. Sep. Purif. Technol..

[27] Hong C.-E., Lee J.-H., Kalappa P., Advani S.G. (2007). Effects of oxidative conditions on properties of multi-walled carbon nanotubes in polymer nanocomposites. Compos. Sci. Technol..

[28] Aghav R.M., Kumar S., Mukherjee S.N. (2011). Artificial neural network modeling in competitive adsorption of phenol and resorcinol from water environment using some carbonaceous adsorbents. J. Hazard. Mater..

[29] Tariq R., Abatal M., Bassam A. (2022). Computational intelligence for empirical modeling and optimization of methylene blue adsorption phenomena using available local zeolites and clay of Morocco. J. Clean. Prod..

[30] Du J., Shang X., Shi J., Guan Y. (2022). Removal of chromium from industrial wastewater by magnetic flocculation treatment: Experimental studies and PSO-BP modelling. J. Water Process Eng..

[31] Ganesan P., Kamaraj R., Sozhan G., Vasudevan S. (2013). Oxidized multiwalled carbon nanotubes as adsorbent for the removal of manganese from aqueous solution. Environ. Sci. Pollut. Res..

[32] Mohamed I.M.A., Dao V.-D., Yasin A.S., Barakat N.A.M., Choi H.-S. (2017). Design of an efficient photoanode for dye-sensitized solar cells using electrospun one-dimensional GO/N-doped nanocomposite SnO_2_/TiO_2_. Appl. Surf. Sci..

[33] Basahel S.N., Al Thabaiti S.A., Obaid A.Y., Mokhtar M., Salam M.A. (2009). Chemical modification of multi-walled carbon nanotubes using different oxidising agents: Optimisation and characterisation. Int. J. Nanopart..

[34] Pillay K., Cukrowska E.M., Coville N.J. (2009). Multi-walled carbon nanotubes as adsorbents for the removal of parts per billion levels of hexavalent chromium from aqueous solution - ScienceDirect. J. Hazard. Mater..

[35] Ali I., Kuznetsova T.S., Burakov A.E., Burakova I.V., Pasko T.V., Dyachkova T.P., Mkrtchyan E.S., Babkin A.V., Tkachev A.G., Albishri H.M. (2022). Polyaniline Modified CNTs and Graphene Nanocomposite for Removal of Lead and Zinc Metal Ions: Kinetics, Thermodynamics and Desorption Studies. Molecules.

[36] Mohamed I.M.A., Yasin A.S., Liu C. (2020). Synthesis, surface characterization and electrochemical performance of ZnO @ activated carbon as a supercapacitor electrode material in acidic and alkaline electrolytes. Ceram. Int..

[37] Sadri R., Hosseini M., Kazi S.N., Bagheri S., Zubir N., Solangi K.H., Zaharinie T., Badarudin A. (2017). A bio-based, facile approach for the preparation of covalently functionalized carbon nanotubes aqueous suspensions and their potential as heat transfer fluids. J. Colloid Interface Sci..

[38] Zhao W., Wang M., Yang B., Feng Q., Liu D. (2022). Enhanced sulfidization flotation mechanism of smithsonite in the synergistic activation system of copper–ammonium species. Miner. Eng..

[39] Lemya B., Zohra S.F., Houari S., Esma C.-B., Boumediene B., Ould K.S., Farouk Z. (2021). Removal of Zn(II) and Ni(II) heavy metal ions by new alginic acid-ester derivatives materials. Carbohydr. Polym..

[40] Silvestro L., Ruviaro A.S., de Matos P.R., Pelisser F., Mezalira D.Z., Gleize P.J.P. (2021). Functionalization of multi-walled carbon nanotubes with 3-aminopropyltriethoxysilane for application in cementitious matrix. Constr. Build. Mater..

[41] Al-Wakeel K.Z., Abd El Monem H., Khalil M.M.H. (2015). Removal of divalent manganese from aqueous solution using glycine modified chitosan resin. J. Environ. Chem. Eng..

[42] Li Y., Shang H., Cao Y., Yang C., Feng Y., Yu Y. (2022). Quantification of adsorption mechanisms distribution of sulfamethoxazole onto biochar by competition relationship in a wide pH range. J. Environ. Chem. Eng..

[43] Stafiej A., Pyrzynska K. (2007). Adsorption of heavy metal ions with carbon nanotubes. Sep. Purif. Technol..

[44] Tsai Y.P., Doong R.A., Yang J.C., Chuang P.C., Chou C.C., Lin J.W. (2013). Removal of Humic Acids in Water by Carbon Nanotubes. Adv. Mater. Res..

[45] Özcan A.S., Erdem B., Özcan A. (2005). Adsorption of Acid Blue 193 from aqueous solutions onto BTMA-bentonite. Colloids Surf. A.

[46] Jun L.Y., Karri R.R., Yon L.S., Mubarak N.M., Bing C.H., Mohammad K., Jagadish P., Abdullah E.C. (2020). Modeling and optimization by particle swarm embedded neural network for adsorption of methylene blue by jicama peroxidase immobilized on buckypaper/polyvinyl alcohol membrane. Environ. Res..

[47] Piao Y., Tondare V.N., Davis C.S., Gorham J.M., Walker A. (2021). Comparative Study of Multiwall Carbon Nanotube Nanocomposites by Raman, SEM, and XPS Measurement Techniques. Compos. Sci. Technol..

[48] Jwa B., Xuan G.A. (2020). Adsorption kinetic models: Physical meanings, applications, and solving methods—ScienceDirect. J. Hazard. Mater..

[49] Wang J., Guo X. (2022). Rethinking of the intraparticle diffusion adsorption kinetics model: Interpretation, solving methods and applications. Chemosphere.

[50] Lagergren S.K. (1898). About the theory of so-called adsorption of soluble substances. Sven. Vetenskapsakad. Handingarl.

[51] Ho Y.S., McKay G. (1998). Sorption of dye from aqueous solution by peat. Chem. Eng. J..

[52] Allen S., Mckay G., Khader K. (1989). Intraparticle diffusion of a basic dye during adsorption onto sphagnum peat. Environ. Pollut..

[53] Weber W.J., Morris J.C. (1962). Proceedings of International Conference Water pollution symposium Pergamon. Oxford.

[54] Langmuir I. (1918). The adsorption of gases on plane surfaces of glass, mica and platinum. J. Am. Chem. Soc..

[55] McKay G., Blair H.S., Gardner J.R. (1982). Adsorption of dyes on chitin. I. Equilibrium studies. J. Appl. Polym. Sci..

[56] Hameed B.H., El-Khaiary M.I. (2008). Malachite green adsorption by rattan sawdust: Isotherm, kinetic and mechanism modeling. J. Hazard. Mater..

[57] Freundlich H. (1907). Uber die adsorption in losungen. Z. Für Phys. Chem..

[58] Foo K.Y., Hameed B.H. (2010). Insights into the Modeling of Adsorption Isotherm Systems.

[59] Yulia F., Chairina I., Zulys A. (2021). Multi-objective Genetic Algorithm Optimization with an Artificial Neural Network for CO_2_/CH_4_ Adsorption Prediction in Metal-Organic Framework. Therm. Sci. Eng. Prog..

[60] Khan H., Hussain S., Hussain S.F., Gul S., Ahmad A., Ullah S. (2021). Multivariate modeling and optimization of Cr(VI) adsorption onto carbonaceous material via response surface models assisted with multiple regression analysis and particle swarm embedded neural network. Environ. Technol. Innov..

